# Supplementary material for: The Valence and Spin State Tuning of Iron(II/III) Porphyrazines with Bulky Pyrrolyl Periphery in Solution and Solid State
Source: Molecules. 2022 Nov 13;27(22):7820. doi: 10.3390/molecules27227820 (PMC9695394; doi:10.3390/molecules27227820)
Supplement: Supplementary file 1 [file molecules-27-07820-s001.zip › molecules-1978646-supplementary.pdf]

The Valence and Spin State Tuning of iron(II/III) Porphyrazines with Bulky Pyrrolyl Periphery in Solution and Solid State

Tomasz Koczorowski [1,*], Wojciech Szczolko [1], Pawel Bakun [1], Barbara Wicher [1], Lukasz Sobotta [2], Maria Gdaniec [3], Anna Teubert [4], Jadwiga Mielcarek [2], Ewa Tykarska [1], Jozef Korecki [5], Kvetoslava Burda [6] and Tomasz Goslinski [1]

[1] Chair and Department of Chemical Technology of Drugs, Poznan University of Medical Sciences, Grunwaldzka 6, 60-780 Poznan, Poland
[2] Chair and Department of Inorganic and Analytical Chemistry, Poznan University of Medical Sciences, Rokietnicka 3, 60-806 Poznan, Poland
[3] Faculty of Chemistry, Adam Mickiewicz University, Uniwersytetu Poznańskiego 8, 61-614 Poznan, Poland
[4] Institute of Bioorganic Chemistry, Polish Academy of Sciences, Z. Noskowskiego 12, 61-704 Poznan, Poland
[5] Jerzy Haber Institute of Catalysis and Surface Chemistry, Polish Academy of Sciences, Niezapominajek 8, 30-239 Krakow, Poland
[6] Faculty of Physics and Applied Computer Science, AGH University of Science and Technology in Krakow, al. Mickiewicza 30, 30-059 Krakow, Poland
* Correspondence: tkoczorowski@ump.edu.pl

SUPPORTING INFORMATION

Table of contents

HPLC purity data 2

UV-Vis solvation study 12

Single crystal X-ray structure determination 16

NMR spectra 18

Mössbauer spectroscopy data 22

HPLC purity data

Analytical HPLC was carried out on an Agilent 1200 instrument equipped with a DAD detector. The chromatographic separation was achieved on reverse stationary phase using isocratic or linear gradient conditions in different phases configurations at 25°C.

PORHYRAZNE 5a

Phases configuration 1

Column: *150 mm × 4.6 mm, 5 µm (Eclipse XDB-C18, Agilent)*

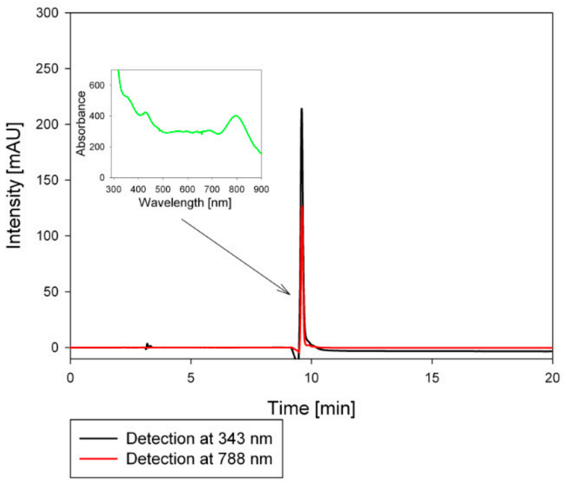

Mobile phase Flow 0.5 ml/min

Time	CH_2Cl_2	MeOH
0	0	100
4	0	100
5	100	0
20	100	0

Detection at λ = 343 nm

Signal	Retention time [min]	Area	Content [%]
1	3.19	18.4	0.84
2	3.33	12.9	0.59
3	9.60	2152.8	98.57

Detection at λ = 788 nm

Signal	Retention time [min]	Area	Content [%]
1	9.61	782.6	100.00

Phases configuration 2

Column: *150 mm × 4.6 mm, 5 µm (Eclipse XDB-C18, Agilent)*

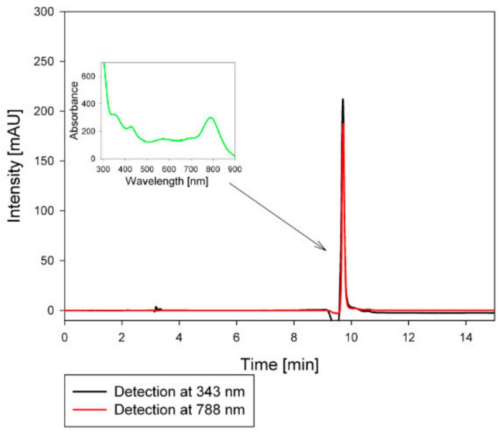

Mobile phase Flow 0.5 ml/min

Time	CH_2Cl_2	MeOH	isopropanol
0	0	100	
4	0	100	
5	100	0	
15	100	0	

Detection at λ = 343 nm

Signal	Retention time [min]	Area	Content [%]
1	3.19	19.8	1.10
2	3.33	14.1	0.78
3	9.71	1773.2	98.12

Detection at λ = 788 nm

Signal	Retention time [min]	Area	Content [%]
1	9.71	1167.7	100.00

Phases configuration 3

Column: *150 mm × 4.6 mm, 5 µm (Eclipse XDB-C18, Agilent)*

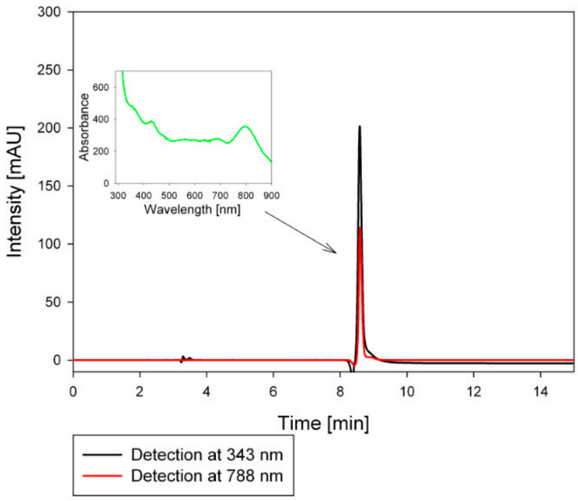

Mobile phase Flow 1.0 ml/min

Time	CH_2Cl_2	MeOH	H_2O
0	0	95	5
4	0	95	5
5	100	0	0
15	100	0	0

Detection at λ = 343 nm

Signal	Retention time [min]	Area	Content [%]
1	3.29	24.3	1.05
2	3.49	22.5	0.97
3	8.58	2265.1	97.97

Detection at λ = 788 nm

Signal	Retention time [min]	Area	Content [%]
1	8.60	772.7	100.00

PORPHYRAZINE 5b

Phases configuration 1

Column: *150 mm × 4.6 mm, 5 µm (Eclipse XDB-C18, Agilent)*

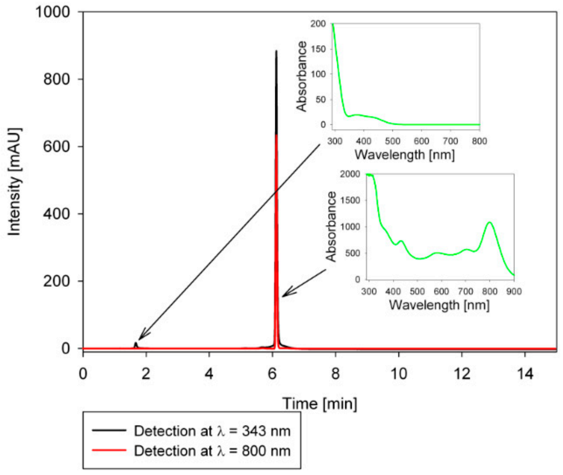

Mobile phase Flow = 1 ml/min

Time	CH_2Cl_2	MeOH
0	0	100
3	0	100
4	100	0
15	100	0

Detection at λ = 343 nm

Signal	Retention time [min]	Area	Content [%]
1	1.67	77.5	2.29
2	5.66	40.5	1.20
3	6.12	3262.8	96.51

Detection at λ = 800 nm

Signal	Retention time [min]	Area	Content [%]
1	6.12	1851.3	100.00

Phases configuration 2

Column: *250 mm × 4.6 mm, 5 µm (Gemini C6-Phenyl, Phenomenex)*

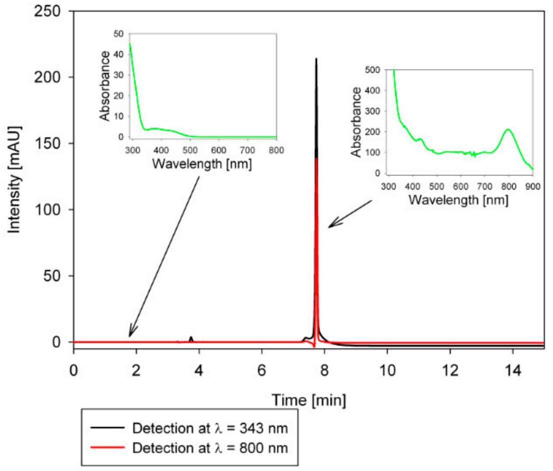

Mobile phase Flow = 1 ml/min

Time	CH_2Cl_2	MeOH
0	0	100
3	0	100
4	100	0
15	100	0

Detection at λ = 343 nm				Detection at λ = 800 nm			
Signal	Retention time [min]	Area	Content [%]	Signal	Retention time [min]	Area	Content [%]
1	3.74	16.5	1.80	1	7.74	470.9	100.00
2	7.40	28.1	3.07				
3	7.73	869.4	95.13				

Phases configuration 3

Column: *250 mm × 4.6 mm, 5 µm (Gemini C6-Phenyl, Phenomenex)*

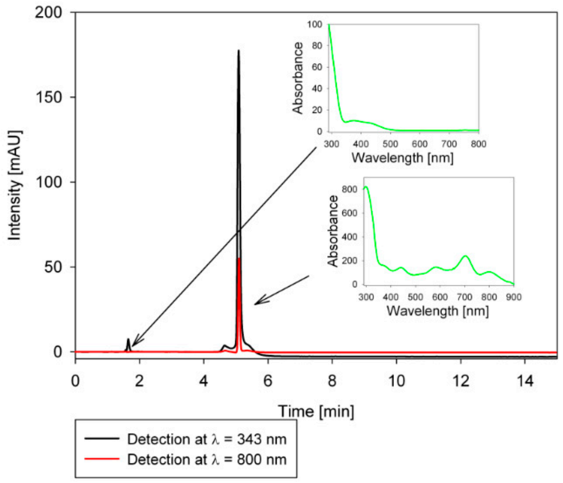

Mobile phase Flow = 1 ml/min

Time	CH_2Cl_2	MeOH	THF
0	0	95	5
3	0	95	5
4	100	0	
15	100	0	

Detection at λ = 343 nm

Signal	Retention time [min]	Area	Content [%]
1	1.65	21.4	1.95
2	4.65	32.6	2.98
3	5.09	1039.3	95.07

Detection at λ = 800 nm

Signal	Retention time [min]	Area	Content [%]
1	5.10	201.7	100.00

PORPHYRAZINE 5c

Phases configuration 1

Column: *150 mm × 4.6 mm, 5 µm (Eclipse XDB-C18, Agilent)*

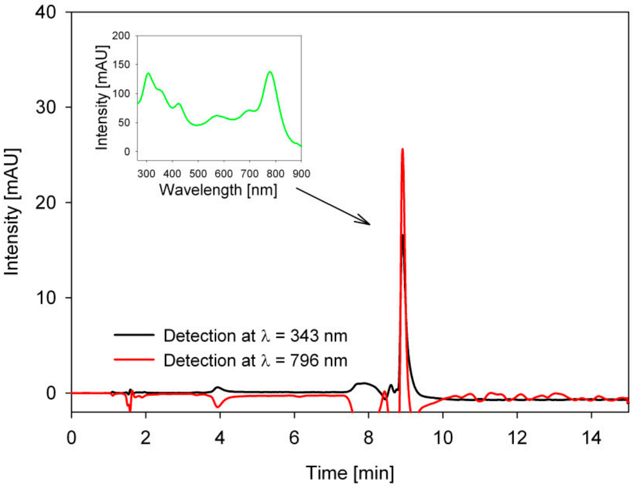

Mobile phase Flow = 1 ml/min

Time	MeOH	CH2Cl2	Isopropanol
0	90	5	5
5	90	5	5
6	5	20	75
15	5	20	75

Detection at λ = 343 nm

Signal	Retention time [min]	Area	Content [%]
1	8.60	11.2	5.42
2	8.91	195.2	94.58

Detection at λ = 796 nm

Signal	Retention time [min]	Area	Content [%]
1	1.63	4.1	1.54
2	8.43	10.0	3.76
3	8.71	3.2	1.20
4	8.91	248.5	93.49

Phases configuration 2

Column: *150 mm × 4.6 mm, 5 µm (Eclipse XDB-C18, Agilent)*

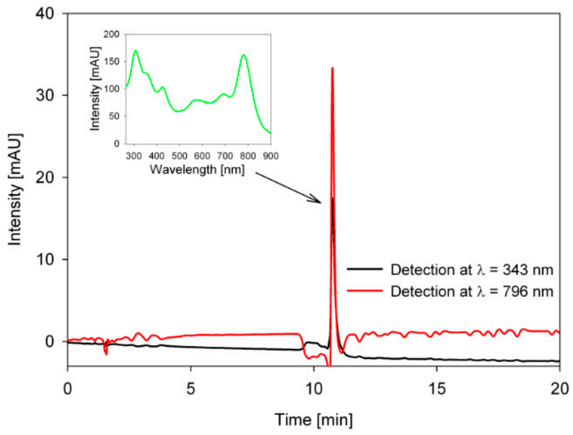

Mobile phase Flow = 1 ml/min

Time	MeOH	CH2Cl2	Isopropanol
0	90	5	5
7	90	5	5
8	10	30	60
20	10	30	60

Detection at λ = 343 nm

Signal	Retention time [min]	Area	Content [%]
1	10.76	199.7	100.00

Detection at λ = 796 nm

Signal	Retention time [min]	Area	Content [%]
1	10.76	272.3	100.00

Phases configuration 3

Column: *150 mm × 4.6 mm, 5 µm (Eclipse XDB-C18, Agilent)*

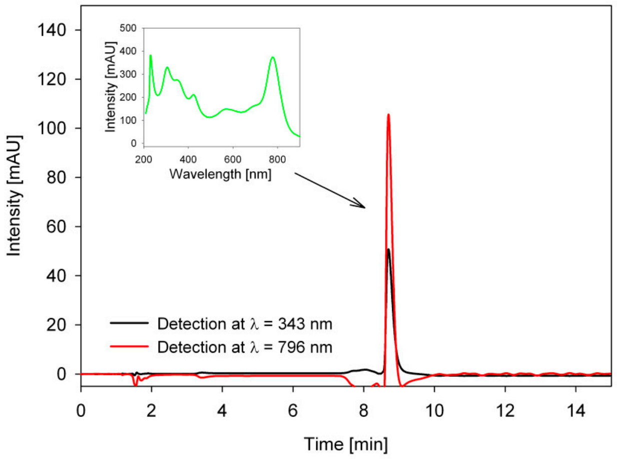

Mobile phase Flow = 1 ml/min

Time	MeOH	CH2Cl2	Isopropanol	CHCl3
0	85	5	5	5
5	85	5	5	5
6	5	20	70	5
20	5	20	70	5

Detection at λ = 343 nm

Signal	Retention time [min]	Area	Content [%]
1	8.70	669.4	100.00

Detection at λ = 796 nm

Signal	Retention time [min]	Area	Content [%]
1	1.63	14.0	1.03
2	8.37	39.9	2.94
3	8.70	1303.6	96.03

UV-Vis solvation studies

Pz 5a

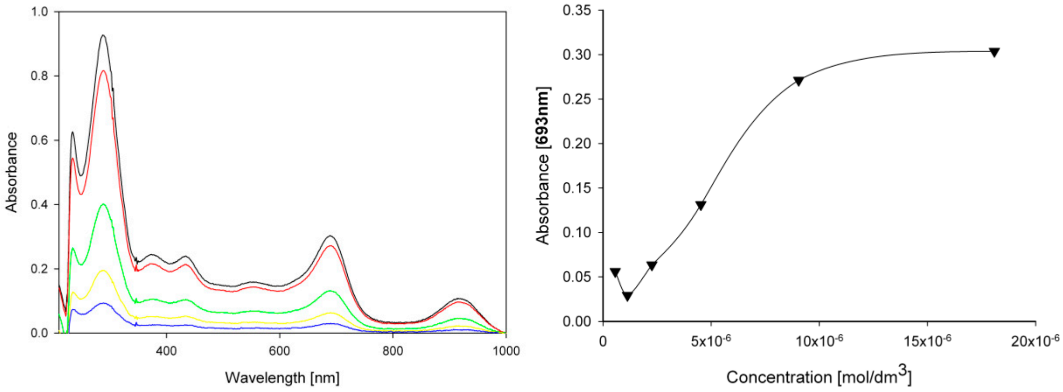

Figure S1. Spectra and correlation between absorbance and concentration at 693 nm for the concentration range $3.62 \cdot 10^{-5} – 1.13 \cdot 10^{-6}$ mol/dm^3 in CH_2Cl_2 for Pz **5a**.

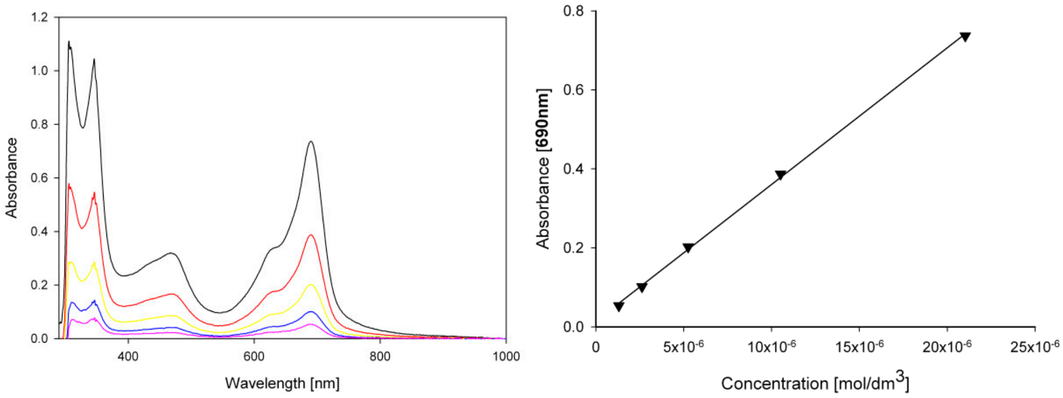

Figure S2. Spectra and linear correlations between absorbance and concentration at 690 nm for the concentration range $4.21 \cdot 10^{-5} – 2.63 \cdot 10^{-6}$ mol/dm^3 in pyridine for Pz **5a**.

Pz 5b

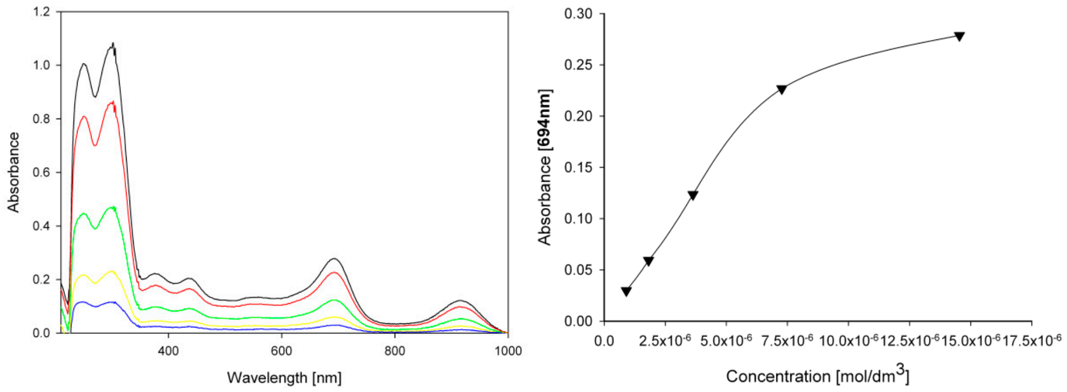

Figure S3. Spectra and correlation between absorbance and concentration at 694 nm for the concentration range 2.91 · 10^{-5} – 1.82 · 10^{-6} mol/dm^3 in CH_2Cl_2 for Pz **5b**.

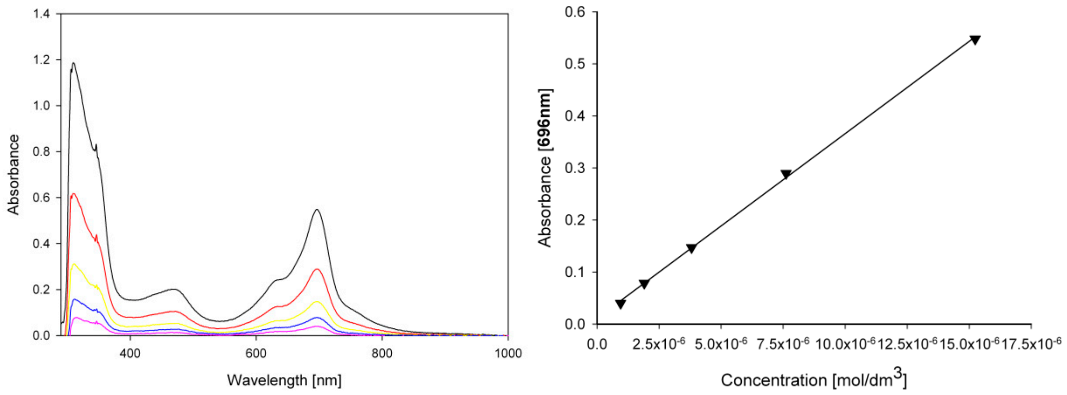

Figure S4. Spectra and linear correlations between absorbance and concentration at 696 nm for the concentration range 3.05 · 10^{-5} – 1.90 · 10^{-6} mol/dm^3 in pyridine for Pz **5b**.

Pz 5c

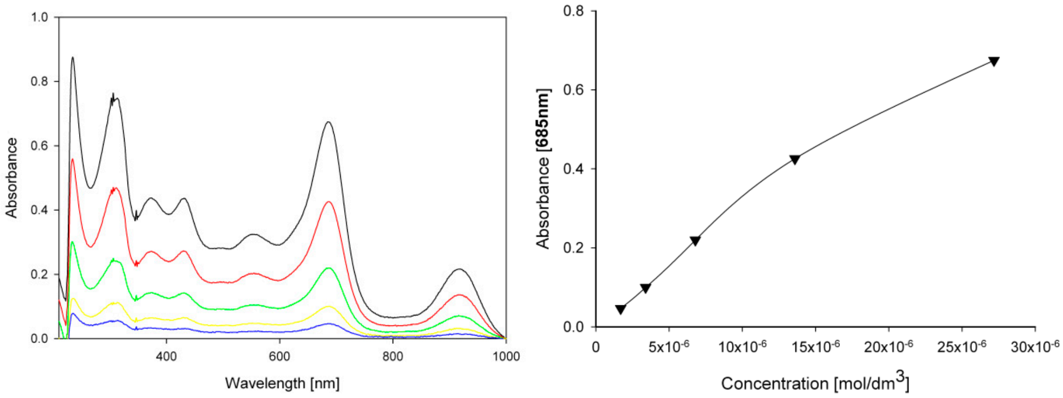

Figure S5. Spectra and correlation between absorbance and concentration at 685 nm for the concentration range 5.44 · 10^{-5} – 3.40 · 10^{-6} mol/dm^3 in CH_2Cl_2 for Pz **5c**.

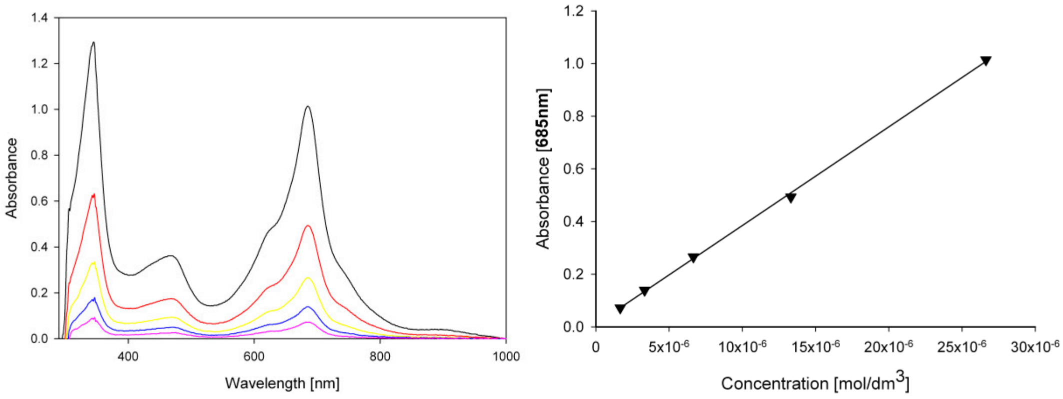

Figure S6. Spectra and linear correlations between absorbance and concentration at 685 nm for the concentration range 5.33 · 10^{-5} – 3.33 · 10^{-6} mol/dm^3 in pyridine for Pz **5c**.

Table S1. Statistical data for solvation study in two solvents

Compound	Solvent	a	b	ta	tb	tα,f	r	n
**Pz 5a**	CH_2Cl_2	Aggregation						
	Pyridine	17304.61	0.01430	68.565	2.618	3.182	1.00	5
**Pz 5b**	CH_2Cl_2	Aggregation						
	Pyridine	17737.32	0.01110	69.361	2.767	3.182	1.00	5
**Pz 5c**	CH_2Cl_2	Aggregation						
	Pyridine	18739.84	0.00997	70.432	1.363	3.182	1.00	5

Single crystal X-ray structure determination

Table S2. X-ray experimental details for porphyrazine **5d**.

	**5d**
empirical formula	$C_{88}H_{72}N_{16}Fe·4(CH_3OH)$ ·unknown solvent
formula weight	1535.61
crystal system	Tetragonal
space group	$I4_1/a$
Temperature (K)	130
a, *b*, *c* (Å)	24.2378 (5), 24.2378 (5), 15.1514 (9)
α, β, γ (°)	90, 90, 90
V (Å^3)	8901.0 (6)
Z	4
radiation type	Mo *K*α
absorption coefficient (mm^{-1})	0.23
reflections collected	32380
reflections observed [$I > 2\sigma(I)$]	3537
R_{int}	0.043
$R[F^2 > 2\sigma(F^2)]$, $wR(F^2)$, S	0.069, 0.188, 1.07
data/parameters/restraints	4530/274/0
largest peak and hole (eA^{-3})	0.51, -0.35

Table S3. The deviation of atoms from least-squares plane of Pz core (* indicates atom used to define a plane)

**5d**	
* -0.004 (0.002) N1A	* 0.004 (0.002) N1A$^{(i)}$
* -0.086 (0.003) C2A	* 0.086 (0.003) C2A$^{(i)}$
* -0.322 (0.003) C3A	* 0.322 (0.003) C3A$^{(i)}$
* -0.371 (0.003) C4A	* 0.371 (0.003) C4A$^{(i)}$
* -0.149 (0.003) C5A	* 0.149 (0.003) C5A$^{(i)}$
* 0.031 (0.002) N1	* -0.031 (0.002) N1$^{(i)}$
* 0.004 (0.002) N1A$^{(ii)}$	* -0.004 (0.002) N1A$^{(iii)}$
* 0.086 (0.003) C2A$^{(ii)}$	* -0.086 (0.003) C2A$^{(iii)}$
* 0.322 (0.003) C3A$^{(ii)}$	* -0.322 (0.003) C3A$^{(iii)}$
* 0.371 (0.003) C4A$^{(ii)}$	* -0.371 (0.003) C4A$^{(iii)}$
* 0.149 (0.003) C5A$^{(ii)}$	* -0.149 (0.003) C5A$^{(iii)}$
* -0.031 (0.002) N1$^{(ii)}$	* 0.031 (0.002) N1$^{(iii)}$
0.0000 (0.0000) Fe1	

Symmetry codes: (i) y-1/4, -x+5/4, -z+5/4; (ii) -y+5/4, x+1/4, -z+5/4; (iii) -x+1, -y+3/2, z

NMR spectra

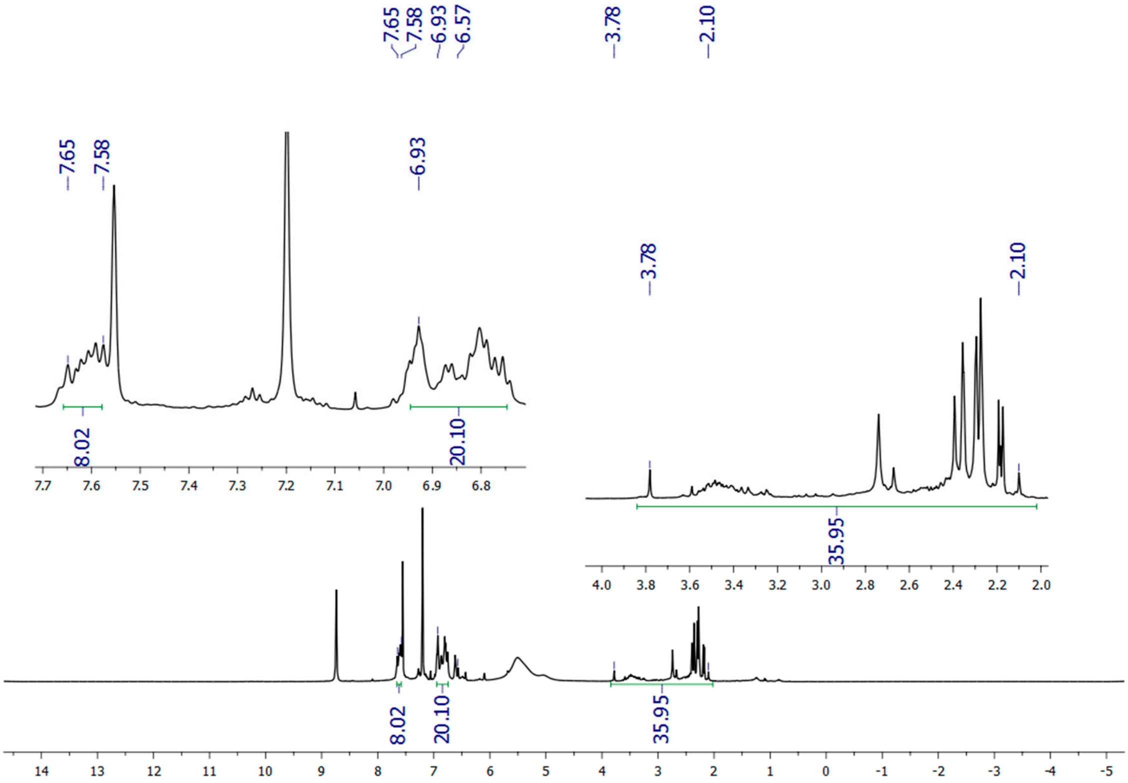

^{1}H NMR [2,7,12,17-Tetrakis(dimethylamino)-3,8,13,18-tetrakis-(2-methyl-5-phenyl-*1H*-pyrrolyl)porphyrazinato]iron(II) (**5a**)

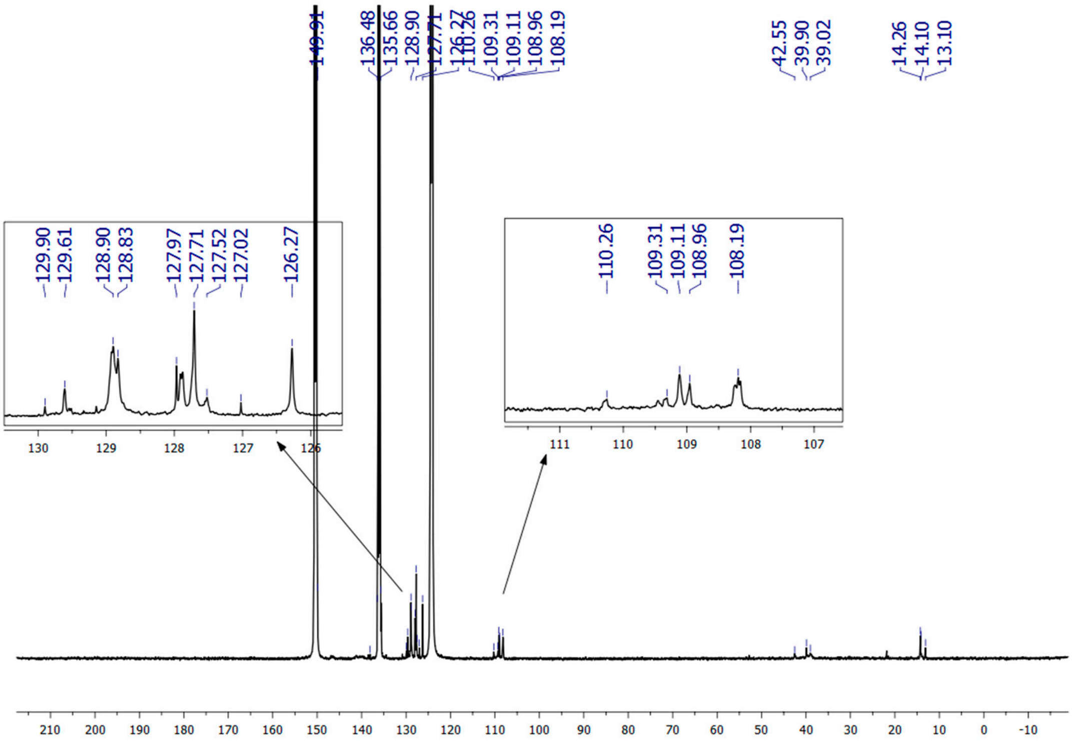

^{13}C NMR [2,7,12,17-Tetrakis(dimethylamino)-3,8,13,18-tetrakis-(2-methyl-5-phenyl-*1H*-pyrrolyl)porphyrazinato]iron(II) (**5a**)

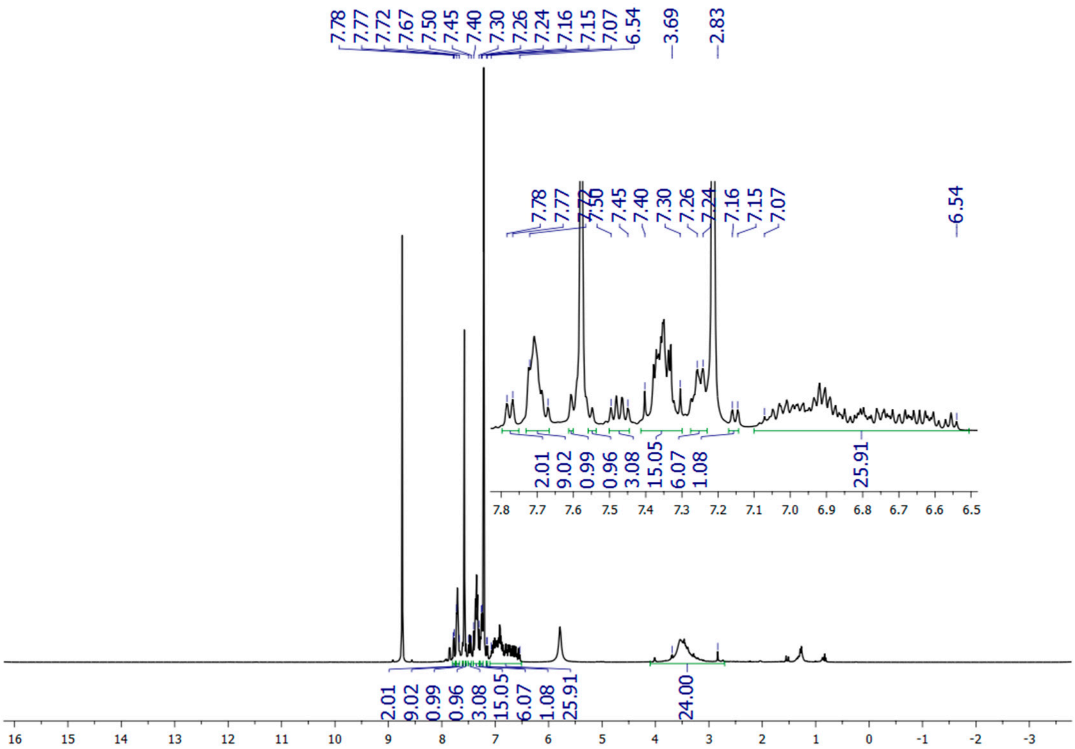

1H NMR [2,7,12,17-Tetrakis(dimethylamino)-3,8,13,18-tetrakis-(2,3,5-triphenyl-*1H*-pyrrol-1-yl)porphyrazinato]iron(II) (**5b**)

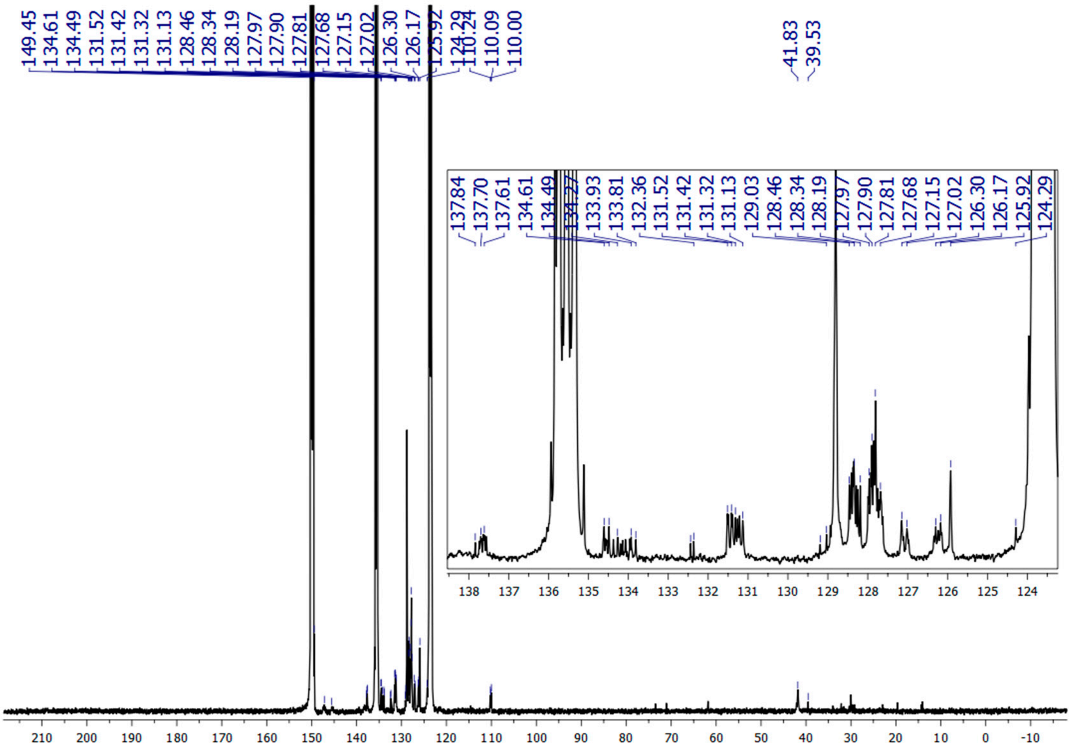

13 C NMR [2,7,12,17-Tetrakis(dimethylamino)-3,8,13,18-tetrakis-(2,3,5-triphenyl-*1H*-pyrrol-1-yl)porphyrazinato]iron(II) (**5b**)

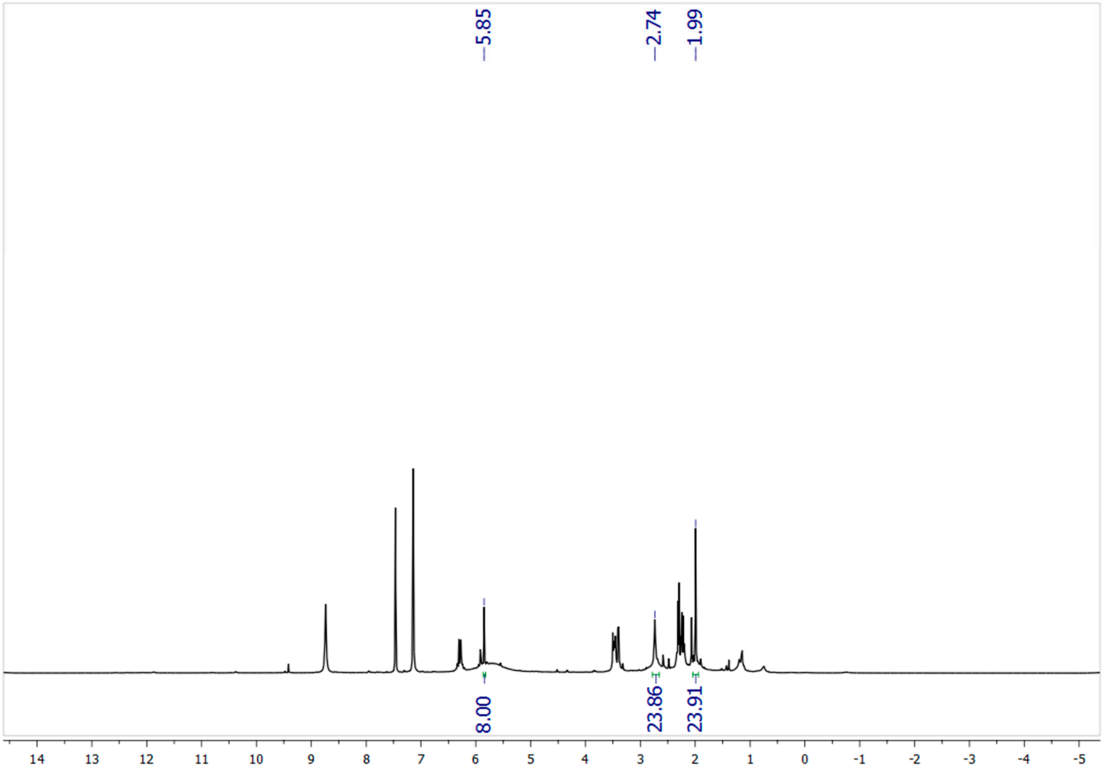

1H NMR [2,7,12,17-Tetrakis(dimethylamino)-3,8,13,18-tetrakis-(2,5-dimethyl-*1H*-pyrrol-1-yl)porphyrazinato]iron(II) (**5c**)

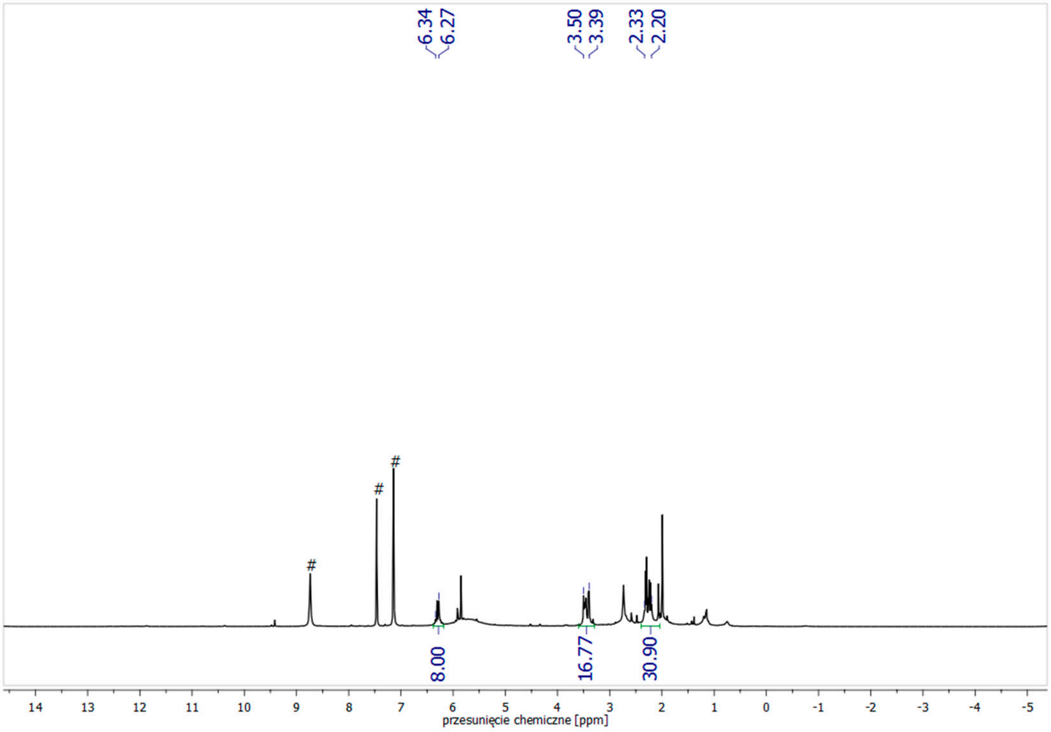

1H NMR [2,7,12,17-Tetrakis(dimethylamino)-3,8,13,18-tetrakis-(2,5-dimethyl-*1H*-pyrrol-1-yl)porphyrazinato] iron(II) (**5c***)

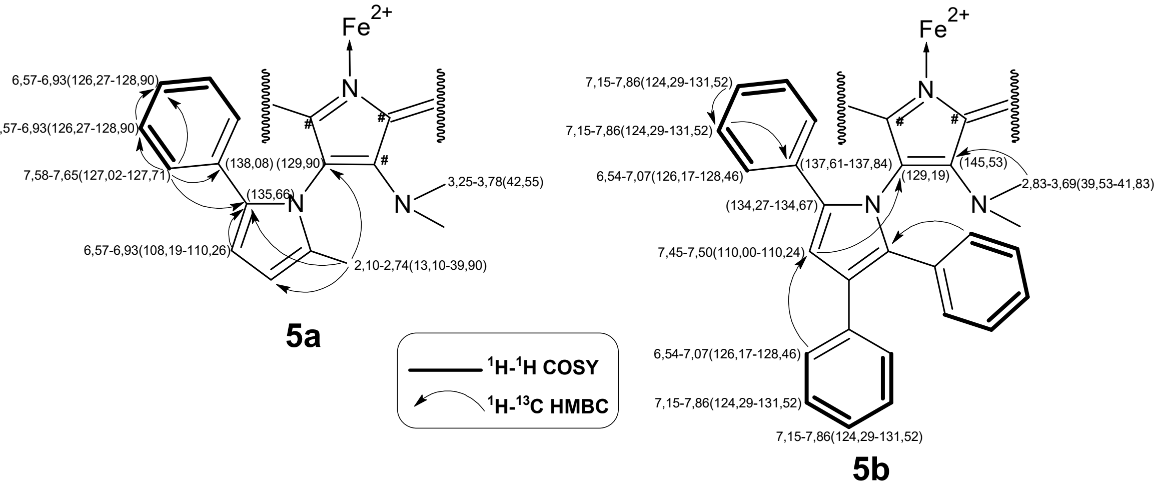

Figure S7. The assignment of ^{1}H and ^{13}C NMR signals of **5a** and **5b** in pyridine-*d*5.

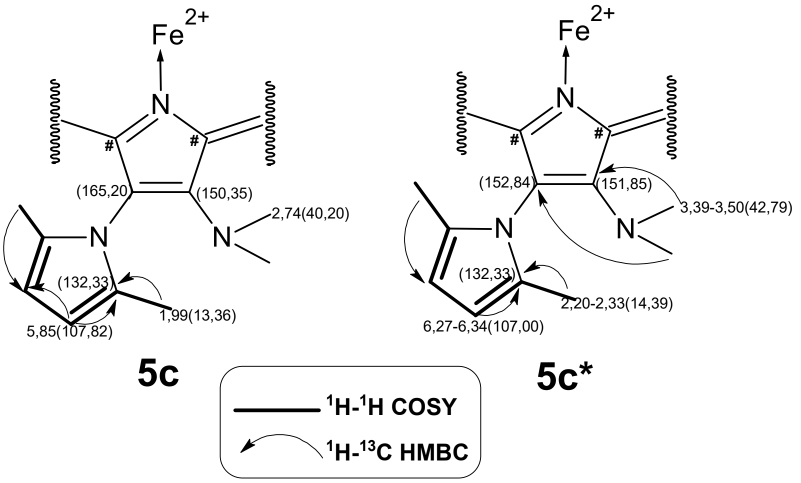

Figure S8. The assignment of ^{1}H and ^{13}C NMR signals of **5c** and **5c*** in pyridine-*d*5.

Mössbauer spectroscopy data

Table S4. Hyperfine parameters (IS-isomer shift, QS-quadrupole splitting) obtained from the theoretical evaluation of the **5c** Mössbauer spectra and contributions of the subsequent components (A).

Component	#1	#2	#3
T = 95 K			
IS [mm/s]	0.17±0.01	0.17±0.03	0.12±0.08
QS [mm/s]	2.89±0.02	3.31±0.12	0.40±0.13
A [%]	78.8±1.5	14.1±1.5	7.1±2.5
T = 135 K			
IS [mm/s]	0.16±0.01	0.14±0.05	0.14±0.07
QS [mm/s]	2.87±0.02	3.37±0.14	0.24±0.12
A [%]	81.3±1.5	10.6±1.5	8.1±2.0
T = 160 K			
IS [mm/s]	0.14±0.01	0.14±0.03	0.10±0.06
QS [mm/s]	2.85±0.03	3.20±0.12	0.45±0.10
A [%]	75.9±1.5	15.9±1.5	8.2±2.0
T = 185 K			
IS [mm/s]	0.15±0.09	0.19±0.06	0.15±0.05
QS [mm/s]	2.85±0.13	3.01±0.31	0.23±0.10
A [%]	68.0±1.5	22.4±1.5	9.7±2.0
T = 295 K			
IS [mm/s]	0.15±0.09	0.08±0.04	0.15±0.10
QS [mm/s	2.57±0.17	2.88±0.14	0.40±0.17
A [%]	27.7±1.5	51.5±1.5	20.8±1.5
T = 304 K			
IS [mm/s]	0.06±0.04	0.08±0.01	0.19±0.01
QS [mm/s]	2.48±0.12	2.76±0.02	0.25±0.04
A [%]	11.6±2.0	64.5±1.5	23.9±1.5

Table S5. Hyperfine parameters (IS-isomer shift, QS-quadrupole splitting) obtained from the theoretical evaluation of the **5a** Mössbauer spectra and contributions of the subsequent components (A).

Component	#1	#2	#3
T = 90 K			
IS [mm/s]	0.20±0.01	0.14±0.01	0.16±0.06
QS [mm/s]	2.71±0.02	3.14±0.12	0.30±0.09
A [%]	42±1.5	51.7±1.5	6.3±2.5
T = 200 K			
IS [mm/s]	0.18±0.01	0.12±0.01	0.23±0.04
QS [mm/s]	2.59±0.05	2.91±0.01	0.25±0.05
A [%]	27.2±1.5	61.0±1.5	11.8±1.5
T = 260 K			
IS [mm/s]	0.15±0.02	0.10±0.01	0.18±0.05
QS [mm/s]	2.56±0.08	2.88±0.04	0.18±0.06
A [%]	28.1±1.5	49.0±1.5	22.9±1.5
T = 300 K			
IS [mm/s]	0.12±0.02	0.08±0.01	0.19±0.01
QS [mm/s]	2.51±0.05	2.88±0.03	0.25±0.02
A [%]	24.1±1.5	39.7±1.5	36.1±1.5

Table S6. Hyperfine parameters (IS-isomer shift, QS-quadrupole splitting) obtained from the theoretical evaluation of the **5b** Mössbauer spectrum and contributions of the subsequent components (A).

T=80K			
Component	#1	#2	#3
IS [mm/s]	0.11±0.03	0.13±0.06	1.02±0.14
QS [mm/s]	2.94±0.05	0.27±0.12	1.55±0.28
A [%]	30.8±3.0	48.8±3.0	20.4±3.0

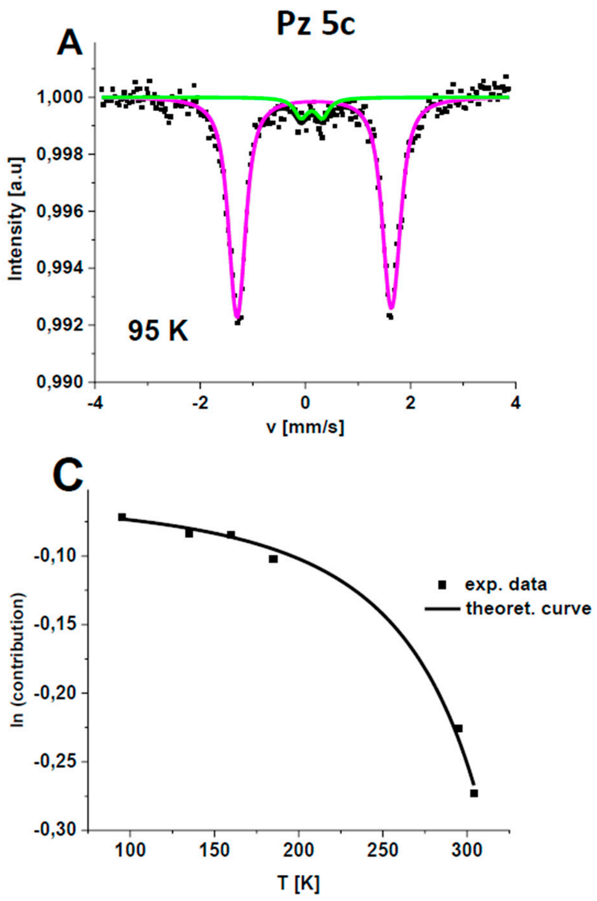

Figure S9. Normalized Mössbauer spectra of Pz **5c** measured at 95 K (A) and Pz **5a** measured at 90 K (B). Symbols represent experimental data and lines theoretical fits. Each subspectrum is marked by different color (component fitted without distribution of the quadrupole splitting - green, component fitted with distribution of the quadrupole splitting - magenta) and black line is a sum of the two subspectra. Temperature dependence of the contribution of the component characterized by high QS with the quadrupole splitting distribution (subspectrum marked by magenta in A and B) for Pz **5c** (C) and Pz **5a** (D). The solid lines in figs. C and D represent theoretical fits obtained by using the simplified Debye model extended for anharmonicity [37,41]. For more details see the main text.
